# Development and testing of the Measure of Innovation-Specific Implementation Intentions (MISII) using Rasch measurement theory

**DOI:** 10.1186/s13012-018-0782-1

**Published:** 2018-06-28

**Authors:** Joanna C. Moullin, Mark G. Ehrhart, Gregory A. Aarons

**Affiliations:** 1 Faculty of Health Sciences, School of Pharmacy and Biomedical Sciences, Curtin University, Building 306, Kent Street, Bentley, Perth, Western Australia 6102 Australia; 2 Child and Adolescent Services Research Center, 3665 Kearny Villa Rd., Suite 200N, San Diego, CA 92123 USA; 3 Department of Psychology, University of Central Florida, Orlando, FL USA; 4 Department of Psychiatry, University of California San Diego, San Diego, USA

**Keywords:** Implementation, Scale development, Rasch, Item response theory, Intentions, Social validity, Program evaluation, Psychometrics, Process assessment, Diffusion of innovation

## Abstract

**Background:**

Implementation is proposed to be a multiphase, multilevel process. After a period of exploration, an adoption decision is made, typically at the upper management or policy level. Nevertheless, movement through each of the subsequent phases of the implementation process involves clinicians or providers at the individual level to adopt the innovation and then change their behavior to use/deliver the innovation. Multiple behavioral change theories propose that intentions are a critical determinant of implementation behavior. However, there is a need for the development and testing of pragmatic measures of providers’ intentions to use a specific innovation or evidence-based practice (EBP).

**Methods:**

Nine items were developed to assess providers’ intentions to use a specific innovation or EBP. Motivational interviewing was the EBP in the study. Items were administered, as part of larger survey, to 179 providers across 38 substance use disorder treatment (SUDT) programs within five agencies in California, USA. Rasch analysis was conducted using RUMM2030 software to assess the items, their overall fit to the Rasch model, the response scale used, individual item fit, differential item functioning (DIF), and person separation.

**Results:**

Following a stepwise process, the scale was reduced from nine items to three items to increase the feasibility and acceptability of the scale while maintaining suitable psychometric properties. The three-item unidimensional scale showed good person separation (PSI = .872), no disordering of thresholds, and no evidence of uniform or non-uniform DIF. Rasch analysis supported the viability of the scale as a measure of implementation intentions.

**Conclusions:**

The Measure of Innovation-Specific Implementation Intentions (MISII) is a sound measure of providers’ intentions to use a specific innovation or EBP. Future evaluation of convergent, divergent, and predictive validity are needed. The study also demonstrates the value of Rasch analysis for testing the psychometric properties of pragmatic implementation measures.

## Background

Delineating the mechanisms by which implementation strategies work is not a simple task. Numerous factors, distributed across levels of the outer system, local setting, and inner organizational context, may influence an implementation strategy’s mechanisms of action [1]. In other words, the mechanisms by which implementation strategies produce results may vary depending on the context and the level of the underlying influences, potentially including those at policy, organization, and/or individual levels. It is therefore important to conduct implementation research using implementation models that hypothesize the direction and influences of such influencing factors (constructs). Subsequently, in order to test these models and mechanisms, it is necessary to have pragmatic, reliable, and valid tools to measure the constructs [2].

Implementation is proposed to be a multilevel, multiphase process that involves policies, organizations, and individual providers. In the present study we use the term “provider” to refer to the individual person, such as a clinician, who may or may not use an innovation in practice [3, 4]. According to the Exploration, Preparation, Implementation, and Sustainment (EPIS) framework, after development or discovery of an innovation, a phase of exploration occurs, concluding with a decision to either adopt or reject the given innovation. If the decision is to adopt, a phase of preparation proceeds, followed by providers beginning to use the innovation (phase of operation/implementation) and ultimately integrating the innovation as routine (sustainment phase) [3]. The adoption decision often is initiated at the upper management or policy level. Yet, the movement through each of the subsequent phases of the implementation process involves individual providers adopting and then using one or more specific innovations or evidence-based practices (EBPs). Because providers may not be involved in the adoption decision, it is critical that, prior to preparation and implementation, organizations understand providers’ attitudes toward the selected innovation and their intentions to use it.

Behavioral change theories, such as the theory of planned behavior [5] and the health belief model [6], posit intentions as a determinant of behavior. In addition, the construct of intentions has been conceptualized as a determinant in implementation theories such as the diffusion of innovations [7]. A systematic review of the relationship between intention and behavior of clinicians suggested a positive relationship between these variables and highlighted the challenge of directly measuring behavior [8], although the degree of concrete evidence of the relationship is debated [8–10]. Techniques are being developed and tested to improve intentions, including action planning for innovations and specific implementation strategies, to promote adoption and use of a given innovation or practice (i.e., change behavior) [11]. In the case of implementation, the sought-after provider behavior is use of the innovation. Past research has supported the relationship between attitudes towards the innovation and such behaviors [12], and in line with theories of behavioral change, we expect that intentions will mediate the relationship between innovation attitudes and participation in the implementation process. Determining providers’ intentions would be a useful proxy outcome with several valuable functions including testing the hypotheses of behavioral change theories, using in implementation simulation studies, and facilitating the tailoring of implementation strategies depending on the measured intentions level.

In 2016, an implementation measure for mental health providers’ intentions to adopt EBP was developed and tested [13]. Despite thorough design and sound psychometric properties, there is a need for an intention measure for use in situations where the decision to adopt a specific innovation or EBP is made at a separate level (i.e., at the upper management or policy level) than where it is being implemented (i.e., the provider level). In addition, there are crucial distinctions between adopting and implementing evidence-based practice generally (i.e., providers being willing to use any EBP) versus the adoption and implementation of a specific practice. Hence, consistent with the reasoning provided by Williams [13], there is need for an intentions scale that is distinct from the adoption decision and that targets providers’ intentions to use a specific innovation. In addition, the movement toward pragmatic measures that are brief, have low burden, are sensitive to change, and have broad applicability suggests the need for a measure that can be tailored for any specific EBP or innovation that is being implemented [2].

Improving the availability of measurement tools with sound psychometric properties has been a priority in implementation science for many years [14]. A number of implementation measures have been developed and tested using the classical test theory (CTT) standards of reliability and validity [15, 16], often using factor analytic approaches in the process. Rasch measurement theory (RMT) offers alternative methods for scale development and evaluation. Both factor analysis and Rasch analysis are classified as latent trait models; however, in the Rasch approach, responses to individual items may be used as indicators of the latent variable, rather than relying on the scale as a whole. In contrast to CTT, in which the focus is on the correlations among the items, the RMT model focuses on the probability of individuals endorsing an item given their responses to other items in the scale [17]. The response to an item is an outcome of the linear probabilistic association between a respondent’s “ability/severity/level” and an item’s “difficulty.” The Rasch model uses a probabilistic Guttmann pattern whereby a hierarchical ordering of items is predicted, such that the probably to endorse higher on the rating scale to an easy item will be greater than the probability to respond higher to a harder item [18]. The probability of a certain response to an item is a “logistic function of the difference between the person’s level of, for example pain, and the level of pain expressed by the item, and only a function of that difference” [19].

RMT was developed within the educational sector, but is now increasingly being used in other fields including health services research [20, 21]. The Rasch model offers some advantages for measure development and testing, such as being able to assess the appropriateness of response options; statistically and graphically evaluate the targeting of the items to the sample; detect items, respondents, or groups of respondents who do not fit the model (e.g., lazy responders or biases for an item among subgroups in the sample); and test the invariance of items. As such, RMT should prove useful in developing a measure of innovation-specific implementation intentions.

The goal of this study was to develop and assess a measure of intentions to use EBP that could be adapted for use with specific EBPs, evidence-informed treatments, or other innovations. To that end, we developed and tested a provider-level measure of implementation intention for a specific innovation using RMT and methods.

## Methods

### Measure

The Measure of Innovation-Specific Implementation Intentions (MISII) was developed following the scale development procedure described by DeVellis which consists of (1) defining what is to be measured (i.e., the construct/latent variable), (2) generating items and the response format using an expert panel, (3) administering items to a sample, and (4) evaluating items and optimizing the scale length [22]. This approach is also consistent with the development of pragmatic measures and in particular having measures with good psychometric properties, high utility, and low burden for administration and scoring.

We convened an expert panel to define the aspects of the intentions construct and to develop items to tap those aspects. The expert panel consisted of two clinical psychologists and two industrial/organizational psychologists, all with extensive knowledge of implementation science and behavioral theory. The aspects characterized by the latent variable, intentions to use an innovation, were considered, and group consensus identified them as plans, desire, and scope. “Plans” pertain to the behavioral aspect of intentions capturing the intensity and definitive commitment a provider has to use the innovation. “Desire” refers to the general motivation or willingness a provider has to use the innovation. “Scope” encapsulates the extent of the innovation a provider aims to deliver. All aspects are thought to derive from the latent variable and therefore to load on one factor, representing a unidimensional latent construct and scale. For each aspect, three items (see Table 1) were developed by the expert panel. A five-point Likert scale was chosen for all items where respondents were instructed to answer regarding the extent to which they intend to use the innovation (motivational interviewing [MI]) on a scale from 0 = not at all to 4 = to a very great extent.

**Table 1:** Original measure of innovation-specific implementation intentions

“Please answer the following questions about the extent to which you intend to use Motivational Interviewing.” 1. I will consider using Motivational Interviewing with new clients. (b) 2. I plan to use Motivational Interviewing with my clients. (a) 3. I am going to apply my training in Motivational Interviewing to address my clients’ needs. (a) 4. I intend to use Motivational Interviewing when appropriate for my clients. (a) 5. Using Motivational Interviewing is a high priority for me. (b) 6. I strive to apply Motivational Interviewing principles in working with my clients. (b) 7. I will use all aspects of Motivational Interviewing with my clients. (c) 8. I will use parts of Motivational Interviewing with my clients. (c) 9. I will use certain Motivational Interviewing strategies with my clients. (c)	

The innovation for the study was Motivational Interviewing

Anchors for the scale were 0 = not at all, 1 = to a slight extent, 2 = to a moderate extent, 3 = to a great extent, 4 = to a very great extent

The dimensions of the latent variable are indicated by (a) plans, (b) desire, and (c) scope

### Context

The study involved analysis of data collected as part of a larger cluster randomized controlled trial of the Leadership and Organizational Change for Implementation (LOCI) implementation strategy [23]. The study was conducted in substance abuse disorder treatment (SUDT) agencies and clinics in California. The study was approved by the University of California San Diego ethics committee as well as the Los Angeles County and San Diego County research ethics committees.

### Participants

Participants for the present study were SUDT providers delivering outpatient or residential services in Southern California. Participants were 61.5% female, with a range of educational levels: 14% master’s degree, 7.8% some graduate work, 26.8% college graduate, 43.0% some college, 3.4% high school diploma, 3.4% General Equivalency Diploma, and 0.6% no high school diploma. Participants’ ethnicity were 36.0% Hispanic or Latino, and race, 60.7% Caucasian, 19.7% Black or African American, 5.1% American Indian or Alaskan Native, 1.7% Asian or Pacific Islander, 1.7% mixed, and 11.2% other. At the time of survey completion, 66.7% of participants were certified or licensed in the addictions field and 12.4% were interns, while 20.3% were not certified or licensed and 0.6% were previously, but not currently, certified or licensed. Characteristics of participants are provided in Table 2. In addition, data on prior to exposure to MI was collected (see Table 3). As compensation for their time completing the survey, participants received a $25 gift card.

**Table 2:** Participant characteristics

Characteristic	*n* (%)
Gender	
Male	68 (38.0%)
Female	110 (61.5%)
Age	
≤ 41 years of age	58 (32.4%)
42–53 years of age	60 (33.5%)
≥ 54 years of age	50 (27.9%)
Ethnicity	
Hispanic or Latino	64 (35.8%)
Race	
Caucasian	108 (60.3%)
American Indian or Alaskan Native	9 (5.0%)
Asian or Pacific Islander	3 (1.7%)
Black or African American	35 (19.6%)
Other	20 (11.2%)
Mixed	3 (1.7%)
Education	
No high school diploma	1 (0.6%)
General Educational Degree (GED)	6 (3.4%)
High school diploma	6 (3.4%)
Some college	77 (43.0%)
College graduate	48 (26.8%)
Some graduate work	14 (7.8%)
Master’s degree	25 (14.0%)
Addiction certification	
Not currently certified or licensed	36 (20.1%)
Currently certified or licensed	118 (65.9%)
Previously certified or licensed	1 (0.6%)
Intern	22 (12.3%)

**Table 3:** Participant exposure to Motivational Interviewing

Characteristic	*n* (%)
Trained in MI	
Yes	132 (73.7%)
No	46 (25.7%)
When were you trained in MI?	
In the past month	13 (7.3%)
In the past 6 months	26 (14.5%)
In the past year	33 (18.4%)
More than a year ago	60 (33.5%)
Unknown	47 (26.3%)
Degree of familiarity with MI principles	
Not at all	6 (3.4%)
To a slight extent	28 (15.6%)
To a moderate extent	92 (51.4%)
To a great extent	42 (23.5%)
To a very great extent	10 (5.6%)
Are you currently using MI with clients?	
Yes	141 (78.8%)
No	37 (20.7%)

### Procedures

At the beginning of the LOCI study, SUDT agency executives were approached by the research team to describe and seek their participation in the LOCI study. Executives, wanting their agency to participate, identified and informed and/or invited suitable work group supervisors (e.g., based on program type, services provided, work group structure) to participate. The research team then followed up with supervisors to provide detailed information about the project and for the research team to further vet the specific clinic or work group. Once determined to fit, and supervisors agreed to participate, they subsequently informed their SUDT providers about the study and the opportunity to participate. Participants had to be at least 18 years of age and employed at one of the participating agencies. All participants were given the opportunity to consent or decline participation in the study as a whole, or to any component of the study. Providers and supervisors received training in MI and were expected to use MI where appropriate with their clients. Provider participants were invited to complete online surveys as part of the LOCI study. The intentions to use MI items were administered at baseline to the first LOCI cohort. In total, 179 substance use disorder treatment (SUDT) providers across 38 SUDT programs within five agencies in California, USA, responded to the survey (86% response rate).

### Data analyses

Data were screened by checking distributional characteristics, the levels of missing data, and for out of range values. Data were fitted to the Rasch-Andrich Rating Scale Model for polytomous response scales [17, 24] using RUMM2030 software [25]. To interpret the fit statistic in Rasch analysis, it is recommended to have 10 participants per item threshold [26], and therefore, 179 providers was an appropriate sample size for the final scale of 3 items and 12 thresholds. Procedures followed were consistent with key Rasch papers [18–20].

Internal consistency reliability of the scale was estimated using the Person Separation Index (PSI), which uses the evaluations of each respondents’ location on the logit scale, rather than the raw score used in Cronbach’s alpha to determine reliability [26]. Interpretation of the PSI is equivalent to Cronbach’s alpha [19].

Three overall fit statistics were considered: two item-person fit statistics and one item-trait interaction statistic. Overall, fit is indicated by a non-significant chi-square statistic (*χ*^2^) (*p* > 0.05 or 0.0056 with Bonferroni adjustment [27]) and fit residual standard deviations of less than 1.4 [28]. In addition, individual item and person fit were determined by a significant *χ*^2^ statistic (*p* < 0.05 or 0.0056 with Bonferroni adjustment), individual fit residuals (between ± 2.5), and item characteristic curves (ICCs). Residuals represent the standardized summation of individual person and item deviations from expected values [19]. ICCs visually indicate fit, including under or over discrimination. A steeper observed curve of respondents compared to the expected model curve indicates over-discrimination and vice versa [28]. In addition, the targeting of the items to the sample was reviewed by assessing the mean location score of respondents.

Sources of deviation from model expectation were analyzed to see if the construction of the scale could be improved, including making the scale more pragmatic by reducing the number of items. A good fitting model shows no disordered response thresholds and no differential item functioning (DIF) for any item. Assessing DIF ensures different groups within the sample (e.g., males versus females or providers within the same agency) did not respond differently to items despite having the same level of intentions.

Finally, a test for violations of local independence (i.e., multidimensionality) was undertaken by a principal component analysis (PCA) of the item residuals, using the pairwise conditional maximum likelihood procedure [26, 29]. A lack of pattern in the residuals supports the assumption of local independence and thus unidimensionality of the scale [30].

The psychometric properties of the scale were also analyzed using common classical test theory methods, the predominant methodology used for scale development in the field of implementation science. Psychometric properties of the scale were preliminarily evaluated using the classical test theory. Reliability was assessed by Cronbach’s alpha and construct validity by exploratory factor analysis (EFA). Data was tested for suitability for factor analysis by ensuring sample size of greater than 10 people per item [16, 31] and the strength of inter-correlations using Bartlett’s test of sphericity [32], Kaiser-Meyer-Olkin (KMO) measure of sampling adequacy [33, 34], and individual measures of sampling adequacy (MSA). Exploratory factor analysis was conducted using principal axis factoring (PAF), with the number of factors based on Kaiser Criterion, Catell’s scree test, and Horn’s parallel analysis [35]. To allow for the possibility of correlations between factors, an oblique rotation (Oblimin) was used.

## Results

The data were verified as suitable for factor and Rasch analysis. There was no missing data for any item, and the minimum and maximum response options were endorsed for all items. Each item had a minimum of one coefficient in the correlation matrix above 0.3. Bartlett’s test of sphericity was statistically significant (*p* < 0.001), and the KMO measure (0.885) and MSAs were above the recommended cut-off values. Cronbach’s alpha of the full scale was 0.933, indicating redundancy of items, and consideration should be given to shorten the scale [22].

The full data set for the nine items were exported to RUMM 2030 for Rasch analysis. The scale showed very good internal consistency with a PSI of 0.891 or 0.898 with extremes included (people that responded all 0 or all 4) [22]. The total chi-square item-trait interaction statistic (35.9674) was significant (*p* < 0.05 or 0.0056 with Bonferroni adjustment), and the fit residual standard deviations for items (2.438) and for persons (1.807) exceeded the recommended value of 1.4, all indicating misfit or deviation from the Rasch measurement model. As per CTT preliminary assessment, evaluation of individual item fit revealed problems with items 8 and 9, which had fit residuals above + 2.5 and a probability value less than the adjusted alpha value (0.0056). Disordered thresholds were not revealed indicating the suitability of the response options. Examination of the ICCs indicated items 2 and 3 were very slightly over-discriminating while items 8 and 9 were under-discriminating. Inspection of individual person fit revealed six people with fit residuals greater than + 2.5. In each case, responses to item 8 were 0, while all other items were endorsed high. Other discrepancies were seen with items 9, 4, and 6. Finally, the scale did not meet the standards for unidimensionality with 7.82% of cases having statistically different scores for the two subtests of items.

**Fig. 1:**
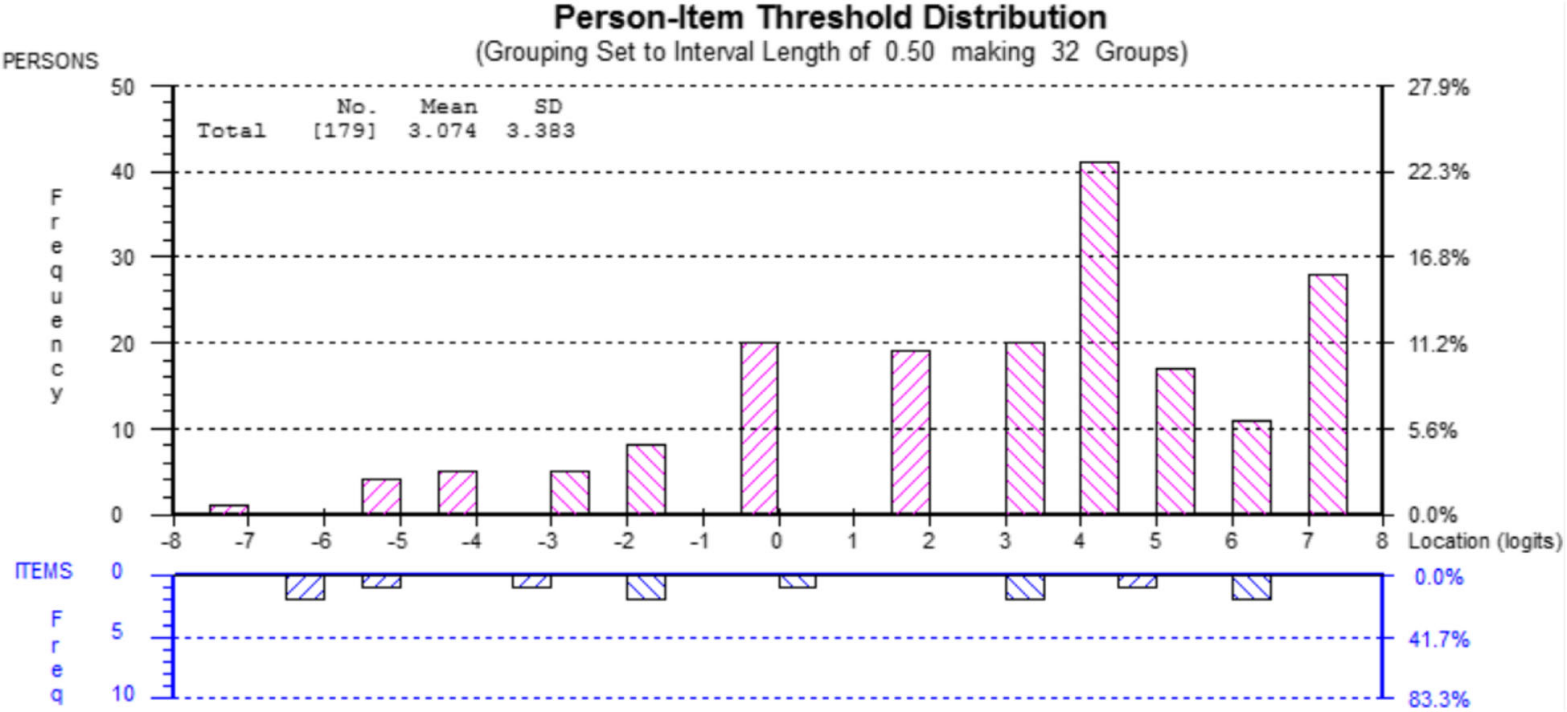
Targeting map for the three-item MISII (*n* = 179)

To resolve issues with model fit, items 8 and 9 were sequentially deleted based on their large fit residuals and their large and significant chi-square statistics. The aspect of intentions covered in the items (scope) was covered in item 7; therefore, removal of the items would not hinder measurement coverage of the latent variable. After the removal of these two items, a unidimensional, seven-item scale resulted in an overall fit to the Rasch model, aside from slight individual item misfit (individual item fit residual − 0.9, SD 3.023). To advance a more pragmatic scale that would reduce burden, a stepwise procedure of item removal was followed (Table 4). Item removal was based on item fit, ensuring a distribution of item difficulties across the latent trait continuum, and ensuring measurement of all aspects of intentions were maintained (Table 5). The targeting map illustrated the items, and thresholds spanned the range of person scores (Fig. 1).

**Table 4:** Summary of results of Rasch analysis of EBP-specific implementation intentions scale

	Analysis	Overall model fit	Item fit residual mean (SD)	Person fit residual mean (SD)	PSI^a^	% Sig. *t* tests^b^
1	Items 1–9	*χ*^2^ = 35.96, *df* = 9, *p* = 0.000	− 0.177 (2.438)	− 0.843 (1.807)	0.898	7.82% *CI*: 0.046 to 0.110
2	Items 1–8	*χ*^2^ = 56.89, *df* = 8, *p* = 0.000	− 0.646 (3.186)	− 0.701 (1.614)	0.898	8.38% *CI*: 0.052 to 0.116
3	Items 1–7	*χ*^2^ = 19.07, *df* = 7, *p* = 0.008	− 0.900 (3.023)	− 0.739 (1.387)	0.909	5.59% *CI*: 0.024 to 0.088
4	Items 1–3, 5–7	*χ*^2^ = 7.50, *df* = 6, *p* = 0.277	− 0.879 (2.374)	− 0.653 (1.209)	0.914	6.70% *CI*: 0.035 to 0.099
5	Items 1–2, 5–7	*χ*^2^ = 6.76, *df* = 5, *p* = 0.239	− 0.578 (1.766)	− 0.619 (1.136)	0.895	6.70% *CI*: 0.035 to 0.099
6	Items 2, 5, 6, 7	*χ*^2^ = 3.20, *df* = 5, *p* = 0.526	− 0.372 (1.158)	− 0.654 (1.100)	0.870	5.59% *CI*: 0.024 to 0.088
7	Items 2, 5, 7	*χ*^2^ = 2.38, *df* = 3, *p* = 0.497	− 0.136 (0.66)	− 0.392 (0.73)	0.872	4.47%

*SD* standard deviation, *χ*^*2*^ chi-square, *df* degrees of freedom, *p* probability, *PSI* person separation index, *CI* confidence interval

^a^PSI with extremes included

^b^Confidence interval only reported if the % value exceeds 5%

**Table 5:** Item location and threshold values (*n* = 150)

Item	Location	SE	Residual	DF	Chi-square statistic	DF	Probability
2	− 1.030	0.166	− 0.696	96.33	7.569	2	0.023
5	0.608	0.156	− 0.301	96.33	4.849	2	0.088
7	0.423	0.157	0.590	96.33	2.484	2	0.289

Outliers/extreme cases *n* = 29 not included

No location values with significant deviations (*p* < 0.001), item fit residuals ±2.5, or reverse thresholds

The final solution was a three-item scale (Table 6) with no misfit to the Rasch measurement model and a PSI of 0.872 (with extremes included), indicating very good internal consistency [22]. Correlation between items was below 0.3 indicating there was no response dependency. In addition, no uniform or non-uniform DIF was found across SUDT agencies, teams, gender, race, ethnicity, certification level, or degree of MI familiarity. Independent *t* tests comparing person trait estimates on the two most divergent items (item 5 and item 7) showed a significant difference (*p* < 0.05) in scores for only 8 of the 179 *t* tests (4.47%), providing support of the unidimensionality of the scale [36].

**Table 6:** Measure of Innovation-Specific Implementation Intentions (MISII)

Please answer the following questions about the extent to which you intend to use [EBP/innovation]						
		Not at all	To a slight extent	To a moderate extent	To a great extent	To a very great extent
1	I plan to use [EBP/innovation] with my clients	0	1	2	3	4
2	Using [EBP/innovation] is a high priority for me	0	1	2	3	4
3	I will use all aspects of [EBP/innovation] with my clients	0	1	2	3	4

EFA was used to examine the potential latent factor structure represented in the data. The EFA was conducted using PAF with correlated factors (oblique) rotation and suggested a two-factor solution (7 items and 2 items), as did Catell’s scree plot and Horn’s parallel analysis. The two items in the second factor (items 8 and 9), however, exhibited high collinearity (0.891), suggesting high conceptual overlap. Item 9 was selected for removal because of its higher mean value and lower standard deviation compared to item 8. With removal of item 9, a one-factor solution resulted and internal consistency remained extremely high, with a Cronbach’s alpha of 0.935 and 67.07% of variance explained. There was high correlation of item 2 with both item 1 (0.898) and item 3 (0.905), indicating removal of one of the items, as per RMT.

Following the RMT process, the psychometric properties of the resulting three-item scale, were evaluated by CCT, which resulted in a Cronbach’s alpha for the scale of 0.900 and 75.25% of variance in the data being explained.

The sample data showed overall high intentions to implement MI (mean person logit score of 3.074, SD 3.383) with responses skewed to higher response options (Table 7). In total, 28 respondents were located at the extreme of the scale, responding 4 (i.e., highest intentions to use MI) to each item. One way analysis of variance (ANOVA) of total scores revealed a significant effect between agencies (F(4, 174) = 3.35, *p* = 0.011) and sexes (F(1, 176) = 10.37, *p* = 0.002) on the total intention scores. Overall, male providers reported lower intentions (mean total score = 2.081, SD 3.48, *n* = 68) than female providers (mean total score of 3.718, SD 3.18, *n* = 110) to use motivational interviewing.

**Table 7:** Proportions of responses (valid percent) in all five response categories (*n* = 150)

Item	Response category				
	0	1	2	3	4
2	1 (0.67%)	10 (6.67%)	29 (19.33%)	78 (52.00%)	32 (21.33%)
5	3 (2.00%)	15 (10.00%)	58 (38.67%)	62 (41.33%)	12 (8.00%)
7	1 (0.67%)	19 (12.67%)	58 (38.67%)	62 (41.33%)	10 (6.67%)

Outliers/extreme cases *n* = 29 not included

## Discussion

The objective of this paper was to develop and examine psychometric characteristics of a brief and pragmatic measurement instrument to assess an individual provider's intentions to implement a specific innovation. The study resulted in the three-item MISII with one item covering each of the three aspects of intentions: plans, desire, and scope. The unidimensional scale fit the Rasch model and showed good reliability with no disordered thresholds or differential item functioning.

The development of a measure of an individual’s intentions to implement an innovation is important as it provides an easily measured proxy for the ultimate outcome, behavioral change (i.e., use of the innovation). In addition, the MISII will facilitate the examination of mechanisms of behavior change such as those postulated in the Theory or Planned Behavior [37]. For example, such mechanisms may include antecedents including attitudes towards an EBP that in turn influence intentions to use the EBP, which then predict subsequent behaviors of adoption and use of the EBP.

The MISII may be useful in many circumstances and for multiple stakeholders of EBP implementation. Currently, upper level management in both the outer system and inner organizational contexts may be unaware of and thus pay little attention to providers’ intentions to use an EBP prior to making an adoption decision. This may be through the lack of understanding about the difficulty of implementing a new EBP, or the inability to easily gauge intentions. Thus, there is potential for the scale to be used to aid the adoption decision in strategic implementation initiatives. This use is further facilitated as it also fulfills multiple required pragmatic criteria of being important to stakeholders, low in burden for respondents and staff, actionable, and likely sensitive to change. It is also consistent with additional recommended criteria including being broadly applicable, unlikely to cause harm, psychometrically strong, and related to theories or models of implementation.

The scale is likely to be equally useful for outer and inner context leaders, middle management, implementation facilitators, and implementation researchers who all may use the results throughout the implementation process to help select and tailor implementation strategies in situations where organizations support or impose the implementation of a specific EBP. For example, an interesting result from the present study was the analysis of variance revealing that males had significantly lower intentions to implement motivational interviewing across all agencies. As such, gender-specific implementation strategies might be tailored to improve the efficiency of implementation across providers [38]. Assessment using the MISII scale may also be used in health care policy making to gauge the impact of policy directives on clinicians and other service providers. Furthermore, the MISII may be used to identify when and where implementation policy initiatives are required, for example, the provision of additional supports or implementation strategies if intentions are determined to be low or sub-optimal. Further, the person location index can identify individuals across the spectrum of low to high intentions. Providers could therefore be segmented into a number of groups depending on their location on the scale to receive different implementation strategies or different intensities of a strategy [39].

Items were created based on the knowledge and experience of a group of clinical and organizational psychologists, all with significant (i.e., many years and diverse range of clinical and organizational research experience) and relevant (i.e., investigators and clinicians leading or involved in multiple implementation projects) implementation expertise. Ideally in RMT during the qualitative stage, item difficulty would be considered in the process, explicitly creating “easier” and more “difficult” items to cover the full range of the latent variable. The final items of the scale showed fairly good spread across the range of person logit scores of the sample. Furthermore, it allowed for redundant items to be easily removed without affecting the measurement of the latent variable nor the validity of the scale. The resulting scale retained three items, one covering each of the three aspects of intentions.

### Strengths and limitations

The MISII is the only implementation measure we are aware of to be developed and tested using RMT (although one study protocol has been published in which RMT was proposed to measure stages of implementation completion [40]). In this study, the instrument was provided to a group of SUDT providers asking about their intentions to use MI. MI is a widely used EBP and the majority of providers were familiar with, had received some training in, and in many cases reportedly were already using the technique to some degree. In addition, for a number of respondents, it was known that a policy level mandate on using MI was about to be enacted. As such, a more diverse calibration sample and assessment of the scale with individuals/providers from other health and allied health sectors and disciplines, and assessment of mechanisms, are recommended. For example, indicators of provider fidelity to EBPs might help to elucidate links between intentions and quality of evidence-based practice use. Intentions to use an intervention do not necessarily imply that the intent is to use with a high level of fidelity. Indeed, for novices in MI, there is likely some naiveté regarding what high quality or expert delivery of MI entails. External validity of the MISII scale may be enhanced by assessment of different EBPs, different settings, and different demographic profiles of participants to increase the generalizability of the scale.

### Future research

We suggest future studies should examine convergent, divergent, discriminant, and criterion-related (concurrent and predictive) validity of the scale to be conducted. It may be possible to evaluate convergent validity with other measures of intentions and divergent validity with measures such as organizational climate, job satisfaction, organizational commitment, and burnout. Discriminant validity could be assessed by examining correlations with measures of hypothesized non-related constructs. Criterion validity of the MISII may be assessed by examining uptake and use of an innovation such as the number of training sessions attended or the degree to which the selected innovation is used in a counseling session or clinical encounter. Testing of sensitivity or responsiveness of the scale would also be useful. This could occur by using the scale in longitudinal studies at multiple time points and/or before and after an implementation strategy is in place. Finally, additional testing of the scale using other innovations or EBPs is needed to demonstrate that the measurement properties hold as the MISII is adapted for other specific practices (e.g., cognitive-behavioral treatment, clinical guidelines, exercise programs).

## Conclusions

The Measure of Innovation-Specific Implementation Intentions (MISII) performed well based on both RMT and CTT methods and is a reliable measure of providers’ intentions to use a specific EBP or new innovation. The measure can be used in applied settings to better understand and prepare for the implementation process and in research settings to better understand the predictors and outcomes of individuals’ implementation-related behavior. In addition, the study indicates the usefulness of the Rasch method of analysis for testing the psychometric properties of implementation measures; future research should continue to apply RMT techniques to evaluate and develop measures of the implementation process.

## Abbreviations

CTT: Classical test theory
DIF: Differential item functioning
EBP: Evidence-based practice
EFA: Exploratory factor analysis
EPIS: Exploration, Preparation, Implementation, and Sustainment
ICCs: Item characteristic curves
KMO: Kaiser-Meyer-Olkin
LOCI: Leadership and Organizational Change for Implementation
MI: Motivational interviewing
MISII: Measure of Innovation-Specific Implementation Intentions
MSA: Measures of sampling adequacy
PAF: Principle axis factoring
PCA: Principle components analysis
PSI: Person Separation Index
RMT: Rasch measurement theory
SUDT: Substance use disorder treatment
